# An Association of Genotypes and Antimicrobial Resistance Patterns among *Salmonella* Isolates from Pigs and Humans in Taiwan

**DOI:** 10.1371/journal.pone.0095772

**Published:** 2014-04-23

**Authors:** Hung-Chih Kuo, Tsai-Ling Lauderdale, Dan-Yuan Lo, Chiou-Lin Chen, Pei-Chen Chen, Shiu-Yun Liang, Jung-Che Kuo, Ying-Shu Liao, Chun-Hsing Liao, Chi-Sen Tsao, Chien-Shun Chiou

**Affiliations:** 1 Department of Veterinary Medicine, National Chiayi University, Chiayi, Taiwan; 2 National Health Research Institutes, Miaoli, Taiwan; 3 Centers for Disease Control, Taichung, Taiwan; Amphia Ziekenhuis, Netherlands

## Abstract

We collected 110 *Salmonella enterica* isolates from sick pigs and determined their serotypes, genotypes using pulsed-field gel electrophoresis (PFGE), and antimicrobial susceptibility to 12 antimicrobials and compared the data with a collection of 18,280 isolates obtained from humans. The pig isolates fell into 12 common serovars for human salmonellosis in Taiwan; *S*. Typhimurium, *S*. Choleraesuis, *S*. Derby, *S*. Livingstone, and *S*. Schwarzengrund were the 5 most common serovars and accounted for a total of 84% of the collection. Of the 110 isolates, 106 (96%) were multidrug resistant (MDR) and 48 (44%) had PFGE patterns found in human isolates. *S*. Typhimurium, *S*. Choleraesuis, and *S*. Schwarzengrund were among the most highly resistant serovars. The majority of the 3 serovars were resistant to 8–11 of the tested antimicrobials. The isolates from pigs and humans sharing a common PFGE pattern displayed identical or very similar resistance patterns and *Salmonella* strains that caused severe infection in pigs were also capable of causing infections in humans. The results indicate that pigs are one of the major reservoirs to human salmonellosis in Taiwan. Almost all of the pig isolates were MDR, which highlights the necessity of strictly regulating the use of antimicrobials in the agriculture sector in Taiwan.

## Introduction

Non-typhoidal *Salmonella* (NTS) is a zoonotic foodborne pathogen that is found worldwide. It is estimated to cause 93.8 million cases of gastroenteritis and 155,000 deaths each year worldwide and approximately 86% of these cases are the result of foodborne infections [1]. In the United States, NTS is responsible for approximately 1 million episodes of salmonellosis each year among all foodborne illnesses that are caused by 31 known pathogens [2]. Domestic and wild animals are the primary reservoirs of NTS. Food animals, including poultry, pigs, and cattle, are the key reservoirs for human salmonellosis [3]. In recent decades, the antimicrobial resistance of NTS has become a large threat to public health. The emergence of antimicrobial resistance and the transmission of resistant strains from animals to humans are mostly associated with the nontherapeutic use of various classes of antimicrobials in large quantities in food animals [4].

Pulsed-field gel electrophoresis (PFGE) is the gold standard molecular subtyping method in differentiating bacterial strains for disease outbreak investigation and disease surveillance. A number of PFGE protocols are standardized for foodborne bacterial pathogens and have been applied to a national foodborne disease surveillance network in the United States [5] and an expanded global surveillance network [6]. The PFGE data generated using a standardized protocol are comparable among laboratories for outbreak investigation and real time disease surveillance and are useful for tracking reservoirs of *Salmonella* infection in humans. Sandt et al. [7] have demonstrated the application of PFGE data for tracking food animal reservoirs for human infections. By comparing PFGE data and antimicrobial susceptibility patterns of *Salmonella* isolates from humans and food animals, they have shown that chickens are major contributors to human salmonellosis in the northeastern United States.

In Taiwan, the burden of salmonellosis in humans, including the number of episodes, hospitalizations, and deaths, has not yet been systematically investigated. From a previously conducted survey administered by our laboratory, we estimate that a total of at least 10,000 *Salmonella* isolates could be recovered each year in the hospitals across Taiwan, a country with a population of 23 million people. The number of isolates suggests that Taiwan could have more than 200,000 episodes of salmonellosis each year, as estimated based on the figures from the United States [2], [8]. The burden of salmonellosis in Taiwan is attributable not only to high incidence rates but also to the high prevalence of multidrug resistance (MDR, resistance to three or more classes of antimicrobial agents) in *Salmonella* strains. Lauderdale et al. [9] showed that several *Salmonella* serovars obtained in Taiwan between 1998 and 2002 displayed a high prevalence of MDR. A comparison study also indicated that *S*. Typhimurium isolates from Taiwan displayed significantly higher levels of antimicrobial resistance than those from Denmark [10]. In the study, cluster G of which the majority were MDR could be unique to Taiwan. To investigate the epidemiological trend of salmonellosis in Taiwan, the *Salmonella* reference laboratory at Taiwan's Centers for Disease Control (TCDC) has begun to collect *Salmonella* isolates from hospitals since 2004 and has performed serotyping, PFGE genotyping, and antimicrobial susceptibility testing for these isolates. To date, more than 20,000 *Salmonella* isolates have been characterized. A database with a large number of *Salmonella* PFGE and antimicrobial susceptibility data is a useful platform for tracking the animal reservoirs of MDR *Salmonella* strains. In this study, we compared the PFGE and antimicrobial susceptibility patterns of *Salmonella* isolates from pigs and humans to investigate whether pigs are major animal reservoirs for human salmonellosis in Taiwan.

## Materials and Methods

### Bacterial isolates

A total of 110 *Salmonella* isolates were recovered from specimens obtained from sick pigs in central and southern Taiwan between 2011 and 2012. The sick pigs were sent to the Veterinary Teaching Hospital of National Chiayi University for pathological diagnosis and necropsy. No animal was killed for the purposes of this study. The specimens were taken from extraintestinal sites, including abscess, bile, joint fluid, liver, lung, and tonsil samples. Human *Salmonella* isolates were collected from a total of 51 hospitals across Taiwan from 2004 to 2012 in the operation of PulseNet Taiwan, a molecular subtyping system for the surveillance of foodborne diseases run by the TCDC. The human isolates were subjected to routine serotyping and PFGE genotyping for detecting disease clusters. The collection of human isolates was approved by the Institutional Review Board of Centers for Disease Control and the institutional review board waived the need for informed consent. All of the data were managed using the computer software BioNumerics version 6.6 (Applied Maths; Kortrijk, Belgium).

### PFGE genotyping

Isolates were subjected to PFGE analysis using the standardized PulseNet PFGE protocol for the subtyping of *Salmonella* and certain other enterobacteria [11]. PFGE images were digitally recorded in tiff file format using a Kodak EDAS290 System (Eastman Kodak Co, Rochester, NY, USA).

### Antimicrobial susceptibility testing (AST)

*Salmonella* isolates were examined for susceptibility to 12 antimicrobials using the microbroth dilution method and custom-made 96-well Sensititre MIC panels (TREK Diagnostic Systems LTD., West Essex, England). Human isolates collected between 2004 and 2010 were tested at the National Health Research Institute, and human isolates collected in 2011 and 2012 and 110 pig isolates were tested at the TCDC. The test procedure was performed according to the manufacturer's instructions. The 12 tested antimicrobials included ampicillin, cefotaxime, ceftazidime, chloramphenicol, ciprofloxacin, gentamicin, imipenem, nalidixic acid, streptomycin, sulfamethoxazole, tetracycline, and trimethoprim/sulfamethoxazole. The criteria of the Clinical and Laboratory Standards Institute (CLSI) [12] were used to interpret the MIC results of the antimicrobial response except for streptomycin: MIC≧64 µg/ml, MIC = 32 µg/ml, and MIC≦16 µg/ml were used to define resistance, intermediate resistance, and susceptibility, respectively.

### Data analysis

PFGE images were analyzed using the fingerprint analysis software BioNumerics version 6.6 (Applied Maths). A PFGE genotype was defined as a PFGE pattern with one or more differential DNA bands present compared to other genotypes. The PFGE patterns were saved in a *Salmonella* database that also contains information regarding serovar, AST, and demographic data for the isolates. The genetic relatedness among the 110 pig isolates was established by cluster analysis of the PFGE patterns using the Dice coefficients, and the unweighted pair group method with an arithmetic mean algorithm and the optimization value and position tolerance were set at 1.56% and 0.75%, respectively.

## Results

### Comparison of serovars and PFGE genotypes

A total of 18,280 human isolates were collected from hospitals across the country between 2004 and 2012. The isolates belonged to 93 serovars and 2,779 PFGE genotypes. *S*. Enteritidis, *S*. Typhimurium, *S*. Stanley, *S*. Newport, and *S*. Albany were the 5 most common serovars (Table 1), and they accounted for 70% of the entire collection. The 110 pig isolates fell into 12 serovars and 44 PFGE types. All of the 12 serovars were among the 40 most common serovars for the human isolates (Table 1). *S*. Typhimurium, *S*. Choleraesuis, *S*. Derby, *S*. Livingstone var. 14+, and *S*. Schwarzengrund were the 5 most common serovars, accounting for 84% of all of the pig isolates. Of the pig isolates, 48 (44%) had PFGE patterns found in the human isolates. Nineteen (43%) of the 44 PFGE types found in the pig isolates were also detected in the human isolates. Each of the 12 serovars had at least one PFGE type that was also found in the human isolates.

**Table 1:** *Salmonella* isolates from humans and pigs.

	Human		Pig	
Serotype	No. isolates	No. PFGE types	No. isolates (No. shared)	No. PFGE types (No. shared)
Enteritidis	5124	193		
Typhimurium	4184	683	53 (13)	20 (6)
Stanley	1531	113	2 (2)	1 (1)
Newport	1254	228	3 (2)	2 (1)
Albany	722	172	5 (1)	1 (1)
Agona	645	94		
Paratyphi B var. Java	547	113		
Weltevreden	504	217		
Derby	452	99	9 (2)	4 (1)
Bareilly	365	32		
Braenderup	363	52		
Schwarzengrund	325	115	7 (2)	2 (1)
Virchow	295	47		
Choleraesuis	276	63	15 (7)	7 (2)
Hadar	247	49	2 (1)	1 (1)
Potsdam	146	42		
Mbandaka	137	39		
Montevideo	116	23		
Blockley	98	14		
Infantis	81	22		
Anatum	80	25	3 (2)	2 (1)
Itami	62	10		
Typhi	56	35		
Livingstone var. 14+	52	9	8 (8)	2 (2)
London	46	21	2 (2)	1 (1)
Cerro	41	7		
Litchfield	40	14		
Saintpaul	39	18		
IIIa 18:z4,z23:-	30	2		
Kedougou	29	14		
Singapore	24	14		
Dublin	23	4		
Isangi	20	7		
Senftenberg	19	17		
Brunei	18	11	1 (1)	1 (1)
Paratyphi A	16	5		
Uganda	16	6		
Havana	15	7		
Haifa	12	2		
Seremban	12	4		
All 53 serotypes	218	137		
**Total**	**18,280**	2,779	110 (48)	44 (19)

### Antimicrobial resistance in pig isolates

**Figure 1:**
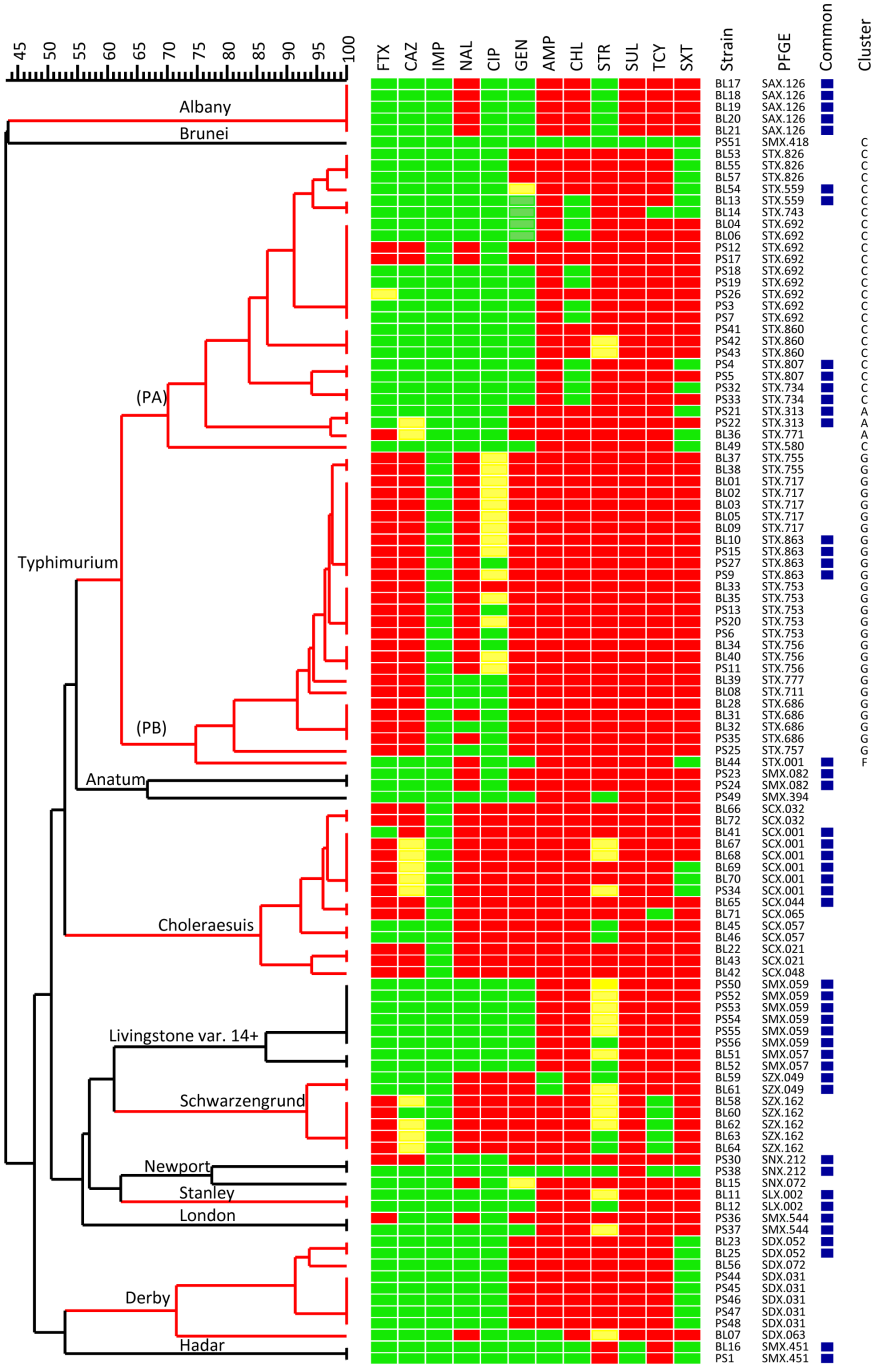
Dendrogram constructed using the PFGE patterns of 110 *Salmonella enterica* isolates from pigs and the corresponding antimicrobial susceptibility patterns to the 12 indicated antimicrobials. The dendrogram was constructed using the unweighted pair group method with an arithmetic mean algorithm. The antimicrobials shown in the sequence are cefotaxime (FTX), ceftazidime (CAZ), imipenem (IMP), nalidixic acid (NAL), ciprofloxacin (CIP), gentamicin (GEN), ampicillin (AMP), chloramphenicol (CHL), streptomycin (STR), sulfamethoxazole (SUL), tetracycline (TCY), and trimethoprim/sulfamethoxazole (SXT). “Resistance” is indicated by a red rectangle, “intermediate resistance” by a yellow rectangle, and “susceptibility” by a green rectangle. PFGE patterns shared by *Salmonella* isolates from human and pig are marked by blue squares. *S.* Typhimurium isolates are indicated with the clusters A, C, G and F, which were designated in a previous study [10].

All but 4 pig isolates were MDR and were resistant to 3 or more classes of antimicrobials (Figure 1). Of the 110 isolates, 48 (44%) were resistant to cefotaxime, 37 (34%) to ceftazidime, 0 (0%) to imipenem, 56 (51%) to nalidixic acid, 23 (21%) to ciprofloxacin, 68 (62%) to gentamicin, 103 (94%) to ampicillin, 94 (85%) to chloramphenicol, 76 (69%) to streptomycin, 107 (97%) to sulfamethoxazole, 101 (92%) to tetracycline, and 83 (75%) to trimethoprim/sulfamethoxazole. *S*. Choleraesuis, *S*. Schwarzengrund, and *S*. Typhimurium isolates displayed the highest resistance profiles. *S*. Choleraesuis isolates were resistant to 8–11 antimicrobials tested (Figure 1). All *S*. Choleraesuis isolates were resistant to ciprofloxacin, and 80% were resistant to cefotaxime. *S*. Schwarzengrund isolates were resistant to 7–8 antimicrobials. All *S*. Schwarzengrund isolates were ciprofloxacin-resistant and 5 of the 7 isolates were resistant to cefotaxime. *S.* Typhimurium isolates fell into two distinct clusters: PA and PB. The majority of isolates in cluster PA were resistant to 3–6 antimicrobials; however, all of the isolates except one in cluster PB were resistant to 8 or more antimicrobials. The majority of isolates in cluster PB were resistant to cefotaxime and ceftazidime and exhibited resistance or reduced susceptibility to ciprofloxacin. By clustering these isolates with 378 *S.* Typhimurium isolates characterized in a previous study [10], all but 3 isolates in cluster PA belonged to the cluster C group, which was defined in the previous study as resistant to ampicillin, streptomycin, sulfonamide, and tetracycline (ASSuT). All but one of the isolates in cluster PB belonged to the previously designated cluster G in which the majority of isolates are resistant to 5 or more antimicrobials. The pig isolates belonging to cluster G were resistant to 8–11 antimicrobials. The isolate BL44 in cluster PB belonged to the previously designated cluster F, which is typically resistant to ASSuT and chloramphenicol (ACSSuT).

### Antimicrobial resistance in the isolates from pigs and humans with common PFGE types

Nineteen of 44 PFGE types found in pig isolates were detected in the human isolates. The 19 PFGE types were found in a total of 2,706 human isolates and accounted for 14.8% of the collection of isolates from hospitals between 2004 and 2012. The resistance patterns were compared between the isolates from pigs and humans with common PFGE types (Table 2). The resistance patterns for the isolates from both sources were either identical or highly similar. Of the 19 common PFGE types, 13 had at least one common resistance pattern found in the isolates from both pigs and humans. The majority of isolates were MDR; 16 PFGE types had consensus patterns with resistance to 4 or more classes of antimicrobials. Seven of 9 *S.* Hadar isolates with the SMX.451 PFGE type from both sources were resistant to only 2 antimicrobials. Seven *S.* Brunei isolates with the SMX.418 PFGE type, 3 *S.* Newport isolates with the SNX.212 PFGE type obtained from humans, and one pig isolate with the SNX.212 PFGE type were pan-susceptible. One *S.* Newport isolate with the SNX.212 PFGE type obtained from pigs exhibited acquired resistance to 9 antimicrobials. When compared to human isolates, some pig isolates displayed acquired resistance to additional antimicrobials. For example, a *S.* Choleraesuis isolate of the SCX.044 type obtained from pigs exhibited additional resistance to cefotaxime and ceftazidime and one of the two *S.* London isolates had developed resistance to 4 additional antimicrobials (Table 2). However, a human *S.* Typhimurium isolate with the STX.313 PFGE type was resistant to 3 additional antimicrobials when compared to the two pigs isolates (Table 2).

**Table 2:** Antimicrobial susceptibility of *Salmonella* isolates with common PFGE types isolated from pigs and humans.

PFGE type	No. isolates (with AST data)		Consensus resistance pattern^1^	FTX	CAZ	IMP	NAL	CIP	GEN	AMP	CHL	STR32	SUL	TCY	SXT
	Human	Pig													
**Albany**	**722**	**5**													
SAX.126	21 (10)	5 (5)	S-S-S-R-S-S-R-R-S-R-R-R	0.0 (0.0)	0.0 (0.0)	0.0 (0.0)	100.0 (100.0)	0.0 (0.0)	0.0 (0.0)	100.0 (100.0)	100.0 (100.0)	20.0 (0.0)	100.0 (100.0)	100.0 (100.0)	100.0 (100.0)
**Anatum**	**80**	**3**													
SMX.082	28 (27)	2 (2)	S-S-S-R-S-S-R-S-S-R-R-R	0.0 (0.0)	0.0 (0.0)	0.0 (0.0)	85.2 (100.0)	0.0 (0.0)	22.2 (100.0)	74.1 (100.0)	44.4 (100.0)	40.7 (100.0)	70.4 (100.0)	74.1 (100.0)	70.4 (100.0)
**Brunei**	**18**	**1**													
SMX.418	6 (6)	1 (1)	S-S-S-S-S-S-S-S-S-S-S-S	0.0 (0.0)	0.0 (0.0)	0.0 (0.0)	0.0 (0.0)	0.0 (0.0)	0.0 (0.0)	0.0 (0.0)	0.0 (0.0)	0.0 (0.0)	0.0 (0.0)	0.0 (0.0)	0.0 (0.0)
**Choleraesuis**	**276**	**15**													
SCX.001	159 (157)	6 (6)	S-S-S-R-R-R-R-R-R-R-R-R	5.1 (83.3)	5.6 (16.7)	0.0 (0.0)	97.5 (100.0)	83.4 (100.0)	61.1 (100.0)	82.2 (100.0)	83.4 (100.0)	81.5 (50.0)	98.1 (100.0)	89.8 (100.0)	77.7 (50.0)
SCX.044	6 (5)	1 (1)	S-S-S-R-R-R-R-R-R-R-R-R	0.0 (100.0)	0.0 (100.0)	0.0 (0.0)	100.0 (100.0)	100.0 (100.0)	100.0 (100.0)	100.0 (100.0)	100.0 (100.0)	100.0 (100.0)	100.0 (100.0)	100.0 (100.0)	100.0 (100.0)
**Derby**	**452**	**9**													
SDX.052	16 (11)	2 (2)	S-S-S-S-S-R-R-R-R-R-R-S	0.0 (0.0)	0.0 (0.0)	0.0 (0.0)	9.1 (0.0)	0.0 (0.0)	72.7 (100.0)	100.0 (100.0)	100.0 (100.0)	100.0 (100.0)	100.0 (100.0)	100.0 (100.0)	0.0 (0.0)
**Hadar**	**247**	**2**													
SMX.451	10 (7)	2 (2)	S-S-S-S-S-S-S-S-R-S-R-S	0.0 (0.0)	0.0 (0.0)	0.0 (0.0)	0.0 (0.0)	0.0 (0.0)	0.0 (0.0)	14.3 (0.0)	14.3 (0.0)	85.7 (100.0)	14.3 (0.0)	85.7 (100.0)	14.3 (0.0)
**Livingstone var. 14+**	**52**	**8**													
SMX.057	2 (1)	2 (2)	S-S-S-S-S-S-R-R-S-R-R-R	0.0 (0.0)	0.0 (0.0)	0.0 (0.0)	0.0 (0.0)	0.0 (0.0)	0.0 (0.0)	100.0 (100.0)	100.0 (100.0)	0.0 (0.0)	100.0 (100.0)	100.0 (100.0)	100.0 (100.0)
SMX.059	13 (5)	6 (6)	S-S-S-S-S-S-R-R-S-R-R-R	0.0 (0.0)	0.0 (0.0)	0.0 (0.0)	0.0 (0.0)	0.0 (0.0)	0.0 (0.0)	100.0 (100.0)	100.0 (100.0)	0.0 (0.0)	100.0 (100.0)	100.0 (100.0)	100.0 (100.0)
**London**	**46**	**2**													
SMX.544	2 (2)	2 (2)	S-S-S-S-S-S-R-R-S-R-R-R	0.0 (50.0)	0.0 (0.0)	0.0 (0.0)	0.0 (50.0)	0.0 (0.0)	0.0 (50.0)	100.0 (100.0)	100.0 (100.0)	0.0 (50.0)	100.0 (100.0)	100.0 (100.0)	100.0 (100.0)
**Newport**	**1,254**	**3**													
SNX.212	4 (3)	2 (2)	S-S-S-S-S-S-S-S-S-S-S-S	0.0 (50.0)	0.0 (50.0)	0.0 (0.0)	0.0 (0.0)	0.0 (0.0)	0.0 (50.0)	0.0 (50.0)	0.0 (50.0)	0.0 (50.0)	0.0 (100.0)	0.0 (50.0)	0.0 (50.0)
**Schwarzengrund**	**325**	**7**													
SZX.049	4 (4)	2 (2)	S-S-S-R-R-R-S-R-S-R-R-R	0.0 (0.0)	0.0 (0.0)	0.0 (0.0)	100.0 (100.0)	100.0 (100.0)	83.3 (100.0)	66.7 (0.0)	100.0 (100.0)	50.0 (0.0)	100.0 (100.0)	100.0 (100.0)	83.3 (100.0)
**Stanley**	**1,531**	**2**													
SLX.002	923 (884)	2 (2)	S-S-S-S-S-S-R-R-S-R-R-R	2.5 (0.0)	2.1 (0.0)	0.0 (0.0)	1.0 (0.0)	0.0 (0.0)	0.3 (0.0)	73.5 (100.0)	99.0 (100.0)	17.5 (0.0)	99.2 (100.0)	99.3 (100.0)	98.5 (100.0)
**Typhimurium**	**4,184**	**53**													
STX.001	1,189 (1,138)	1 (1)	S-S-S-R-S-S-R-R-R-R-R-S	1.1 (0.0)	1.0 (0.0)	0.0 (0.0)	51.0 (100.0)	0.0 (0.0)	0.4 (0.0)	97.0 (100.0)	94.7 (100.0)	95.1 (100.0)	98.3 (100.0)	96.0 (100.0)	2.0 (0.0)
STX.313	1 (1)	2 (2)	S-S-S-S-S-R-R-R-R-R-R-R	100.0 (0.0)	100.0 (0.0)	0.0 (0.0)	100.0 (0.0)	0.0 (0.0)	100.0 (100.0)	100.0 (100.0)	100.0 (100.0)	100.0 (100.0)	100.0 (100.0)	100.0 (100.0)	100.0 (50.0)
STX.559	237 (180)	2 (2)	S-S-S-S-S-S-R-S-R-R-R-S	0.0 (0.0)	0.0 (0.0)	0.0 (0.0)	1.1 (0.0)	0.0 (0.0)	0.6 (0.0)	95.6 (100.0)	1.1 (50.0)	95.6 (100.0)	95.0 (100.0)	97.8 (100.0)	1.1 (0.0)
STX.734	40 (2)	2 (2)	S-S-S-S-S-S-R-S-R-R-R-S	0.0 (0.0)	0.0 (0.0)	0.0 (0.0)	0.0 (0.0)	0.0 (0.0)	0.0 (0.0)	100.0 (100.0)	0.0 (0.0)	100.0 (100.0)	100.0 (100.0)	100.0 (100.0)	0.0 (50.0)
STX.807	44 (1)	2 (2)	S-S-S-S-S-S-R-S-R-R-R-S	0.0 (0.0)	0.0 (0.0)	0.0 (0.0)	0.0 (0.0)	0.0 (0.0)	0.0 (0.0)	100.0 (100.0)	0.0 (0.0)	100.0 (100.0)	100.0 (100.0)	100.0 (100.0)	0.0 (50.0)
STX.863	1 (1)	4 (4)	R-R-S-R-S-R-R-R-R-R-R-R	100.0 (100.0)	100.0 (100.0)	0.0 (0.0)	100.0 (100.0)	0.0 (0.0)	100.0 (100.0)	100.0 (100.0)	100.0 (100.0)	100.0 (100.0)	100.0 (100.0)	100.0 (100.0)	100.0 (100.0)
Total	2,706 (2,445)	110 (110)		1.8 (25.0)	1.6 (14.6)	0.0 (0.0)	32.4 (45.8)	5.7 (18.8)	5.3 (43.8)	86.7 (87.5)	87.5 (81.25)	64.8 (52.1)	97.5 (93.8)	96.4 (95.8)	43.5 (68.8)

1 The antimicrobials in the sequence (FTX-CAZ-IMP-NAL-CIP-GEN-CAMP-CHL-STR32-SUL-TCY-SXT) are cefotaxime (FTX), ceftazidime (CAZ), imipenem (IMP), nalidixic acid (NAL), ciprofloxacin (CIP), gentamicin (GEN), ampicillin (AMP), chloramphenicol (CHL), streptomycin (STR32), sulfamethoxazole (SUL), tetracycline (TCY), and trimethoprim/sulfamethoxazole (SXT). R, resistance; I, intermediate resistance; S, susceptibility.

## Discussion

Our data show that all of the 12 serovars identified among the 110 pig *Salmonella* isolates are prevalent in the human clinical isolates obtained in Taiwan between 2004 and 2012. Of the 110 isolates from pigs, 44% shared PFGE patterns with the human isolates. The 19 common PFGE patterns are found in 14.8% of human isolates. Almost all of the pig *Salmonella* isolates were MDR and the resistance patterns for the isolates with common PFGE patterns from pigs and humans were either identical or highly similar. These data suggest a close relationship between *Salmonella* strains from pigs and humans. In this study, only a small number of isolates from pigs were compared. If more pig isolates were investigated, more common PFGE patterns would be identified and the ratio of *Salmonella* strains from pigs associated with human salmonellosis would be higher.

Sandt et al. [7] compare the 20 most common human *Salmonella* PFGE patterns and antimicrobial susceptibility patterns in *Salmonella* isolates from cattle, chickens, pigs, and turkeys to investigate the relationship between *Salmonella* causing human infections and their food animal reservoirs. Their findings indicate that chickens are major contributors to human salmonellosis in the northeastern United States. The data also show that the *Salmonella* isolates have a low MDR rate. In another study, we compared the genotypes and antimicrobial susceptibility patterns in *Salmonella* isolates from chickens, pigs, ducks, geese, and turkeys with those from humans. The study shows that pigs and chickens rank as the first and the second major contributors of MDR *Salmonella* with regard to human infections in Taiwan (unpublished data).

Astonishingly, 96% of the 110 pig *Salmonella* isolates were MDR, and 47% of them were resistant to 8 or more of the 12 antimicrobials tested. Some of the MDR strains have been the major causes of human infections in Taiwan. Strains of *S.* Choleraesuis are one of them. *S.* Choleraesuis is host-adapted to swine, and it rarely causes infection in humans [8]; however, it is extremely invasive and results in high mortality rates in humans [13], [14]. The epidemiologic profiles are quite different in some Asian countries. *S.* Choleraesuis was the 11^th^ most common serovar responsible for human infections in Thailand between 1993 and 2002 [15]. In Taiwan, *S.* Choleraesuis was the second most common serovar in human *Salmonella* isolates in the 1990s [14]. Our surveillance data indicate that *S.* Choleraesuis was still highly prevalent in Taiwan in 2004: it accounted for 4.4% of the collection and ranked as the 5^th^ most common serovar that year. The proportion of this serovar has decreased since 2005: it accounted for only 1.5% of the collection between 2004 and 2012 (Table 1). One previous study conducted by our laboratory indicated that the *S.* Choleraesuis epidemic in humans and swine was likely caused by a common clone and that the majority of *S.* Choleraesuis isolates were MDR [16]. Fluoroquinolone-resistant *S.* Choleraesuis strains, which most likely evolved from the major clone, emerged in 2000 and caused widespread infections among humans and pigs [16], [17]. Resistance to extended-spectrum cephalosporins has been frequently detected in *S.* Choleraesuis. The increase of the resistance was likely due to the transmission of the *ampC* gene *bla*_CMY-2_ [18], [19].

*S.* Typhimurium is the second most common serovar among the isolates collected from hospitals in Taiwan between 2004 and 2012 (Table 1). A previous study showed that human *S.* Typhimurium isolates from Taiwan displayed high resistance rates to nalidixic acid, gentamicin, chloramphenicol, and trimethoprim/sulfamethoxazole, and extremely high resistance rates to ampicillin, streptomycin, sulfamethoxazole, and tetracycline [10]. When compared to the isolates collected from Denmark, the isolates from Taiwan display significantly higher resistance rates to 11 of the 12 tested antimicrobials. In that study, 7 genetic clusters (A–G) were designated, and a high percentage of the isolates in genetic clusters C, F and G were MDR. In the present study, the pig *S.* Typhimurium isolates fall into two distinct clusters: PA and PB (Figure 1). The isolates are compared with the 378 isolates assayed by the previous study. By clustering analysis of the PFGE patterns, all but 3 isolates in cluster PA are grouped tightly with those in cluster C, and these strains are typically resistant to ASSuT (data not shown). The ASSuT-resistant clone, which likely originated in pigs [20], was first identified in Italy and has spread globally [21], [22]. The majority of ASSuT-resistant human isolates belonged to the PFGE types STX.559 and STX.734. In Taiwan, the STX.559 type was first detected in 2008 and has now become prevalent in Taiwan. STX.734 was predominant in the ASSuT-resistant isolates from Denmark and was first detected in 2010 in Taiwan. STX.734 is now common in Taiwan. In this study, both STX.559 and STX.734 were detected in pig isolates. Obviously, the ASSuT-resistant clone has expanded, and some strains have acquired additional resistance to cefotaxime, ceftazidime, nalidixic acid, gentamicin, chloramphenicol, and trimethoprim (Figure 1). All but one isolate in cluster PB are clustered with those isolates found in cluster G which are characterized as bearing resistance to more than 5 antimicrobials and are likely unique to Taiwan. In this study, all of the pig isolates belonging to cluster G are resistant to 9–11 of the antimicrobials tested (Figure 1). The pig isolates in cluster G are very closely related. One PFGE type (STX.863) was detected in human isolates indicating that pigs are likely the reservoir of clone G. However, we also identified strains of clone G isolated from chickens, turkeys, and geese (data not shown). The strains present in turkeys and geese have similar resistance patterns to those clone G strains isolated from pigs and humans. It is very unfortunate that this MDR clone has become widespread among the major food animal stocks in Taiwan.

*S.* Schwarzengrund is the 12^th^ most common serovar and accounts for 1.8% of the collection of human isolates (Table 1). Poultry and swine are the primary reservoirs of this serovar [15], . *S.* Schwarzengrund is one of the most invasive serovars, it, together with *S.* Choleraesuis, *S.* Typhimurium, and *S.* Enteritidis, accounted for approximately 70% of invasive salmonellosis in Taiwan between 1998 and 2002 [9]. International spread of MDR *S.* Schwarzengrund has become a great concern issue. Transmission of this serovar across country borders most likely occurs through international trade of food products, mainly poultry meat and pork [26]. A *Salmonella* surveillance study performed in Taiwan from 2001 to 2005 showed that *S.* Schwarzengrund and *S.* Albany were the most prevalent serovars found in chicken meat obtained from wet markets, and all of the chicken meat isolates and human isolates included for comparison were MDR and displayed a high prevalence of resistance to ampicillin, gentamicin, kanamycin, streptomycin, tetracycline, nalidixic acid, trimethoprim-sulfamethoxazole, and chloramphenicol [23]. Studies by comparing the genotypes of isolates from chickens, turkeys, pigs, and humans suggest that the transmission of *S.* Schwarzengrund from food products to humans is possible [26], [27]. Our data also show a connection between the *S.* Schwarzengrund MDR isolates from pigs and humans.

*S.* Albany is detected primarily in poultry and not a prevalent serovar among clinical isolates in the USA and Thailand [8], [15], [28], [29]. This serovar was unusually prevalent in Taiwan, and it ranked as the 5^th^ most common serovar for human salmonellosis between 2004 and 2012 (Table 1). Our data indicate that the 15 *S.* Albany isolates of the SAX.126 type from pigs and humans are all resistant to ampicillin, chloramphenicol, sulfamethoxazole, tetracylines, trimethoprim, and nalidixic acid (Table 2). These isolates could carry the *Salmonella* genomic island SGI1-F that confers resistance to the first 5 antimicrobials [30]. Of the 678 human *S.* Albany isolates in our database that contain AST data, 652 (96%) are resistant to the 5 antimicrobials mediated by the presence of SGI1-F and 604 (89%) are also resistant to nalidixic acid. The highly consistent resistance patterns among the isolates from pigs and humans suggest that the *S.* Albany strains circulating in Taiwan could have been derived from clonal expansion of a SGI1-F-carrying strain.

*S.* Livingstone is primarily detected in chicken and swine samples [8], [31], [32]. It was not a prevalent serovar in human salmonellosis in Taiwan between 2004 and 2012 (Table 1), but human infections with this serovar have increased since 2010. The two PFGE types SMX.057 and SMX.059 were found in isolates from both humans and pigs and were first detected in humans in 2010. The incidence of the SMX.059 type significantly increased in 2012. SMX.057 and SMX.059 isolates from both sources are MDR, while the majority of isolates with other PFGE types are pan-susceptible. Thus, pigs could be one of the major reservoirs of MDR *S.* Livingstone for human infections in Taiwan.

*S.* Derby is a prevalent serovar among *Salmonella* isolates obtained from food animals and humans [8], [25], [28], [33]. This serovar displays a high prevalence of MDR. Of the 418 *S.* Derby isolates with AST data in our database, 304 (73%) are MDR and primarily exhibit resistance to sulfamethoxazole, streptomycin, and tetracycline. Approximately 20% to 40% of the isolates have developed additional resistance to ampicillin, chloramphenicol, nalidixic acid, and trimethoprim, and approximately 3% of these isolates are also resistant to cefotaxime, ceftazidime, and gentamicin.

In conclusion, *Salmonella* isolates recovered from sick pigs belonged to 12 frequently identified serovars for human salmonellosis in Taiwan between 2004 and 2012. It is notable that almost all of the pig *Salmonella* isolates are MDR and that 47% are resistant to 8 or more antimicrobials tested. The isolates from pigs and humans with a common PFGE pattern had either identical or very similar resistance patterns. *S.* enterica strains which can cause severe invasive infections in pigs could also be a major cause for human salmonellosis in Taiwan. Thus, pigs could serve as the principal *Salmonella* reservoirs for human salmonellosis. The agriculture sector must face this severe problem and implement effective measures to control *Salmonella* infection in pig farms and strictly regulate the use of antimicrobials.
